# Contribution of oligomerization to the anti-HIV-1 properties of SAMHD1

**DOI:** 10.1186/1742-4690-10-131

**Published:** 2013-11-12

**Authors:** Alberto Brandariz-Nuñez, Jose Carlos Valle-Casuso, Tommy E White, Laura Nguyen, Akash Bhattacharya, Zhonghua Wang, Borries Demeler, Sarah Amie, Caitlin Knowlton, Baek Kim, Dmitri N Ivanov, Felipe Diaz-Griffero

**Affiliations:** 1 Department of Microbiology and Immunology, Albert Einstein College of Medicine, 1301 Morris Park – Price Center 501, Bronx, NY 10461, USA; 2 Center for Drug Discovery, Emory School of Medicine, Atlanta, GA, USA; 3 Department of Biochemistry and Cancer Therapy and Research Center, University of Texas Health Science Center at San Antonio, San Antonio, TX 78229, USA

**Keywords:** SAMHD1, Oligomerization, Tetramer, HIV-1, Restriction, Deoxynucleotides, Nuclease activity

## Abstract

**Background:**

SAMHD1 is a restriction factor that potently blocks infection by HIV-1 and other retroviruses. We have previously demonstrated that SAMHD1 oligomerizes in mammalian cells by immunoprecipitation. Here we investigated the contribution of SAMHD1 oligomerization to retroviral restriction.

**Results:**

Structural analysis of SAMHD1 and homologous HD domain proteins revealed that key hydrophobic residues Y146, Y154, L428 and Y432 stabilize the extensive dimer interface observed in the SAMHD1 crystal structure. Full-length SAMHD1 variants Y146S/Y154S and L428S/Y432S lost their ability to oligomerize tested by immunoprecipitation in mammalian cells. In agreement with these observations, the Y146S/Y154S variant of a bacterial construct expressing the HD domain of human SAMHD1 (residues 109–626) disrupted the dGTP-dependent tetramerization of SAMHD1 *in vitro*. Tetramerization-defective variants of the full-length SAMHD1 immunoprecipitated from mammalian cells and of the bacterially-expressed HD domain construct lost their dNTPase activity. The nuclease activity of the HD domain construct was not perturbed by the Y146S/Y154S mutations. Remarkably, oligomerization-deficient SAMHD1 variants potently restricted HIV-1 infection.

**Conclusions:**

These results suggested that SAMHD1 oligomerization is not required for the ability of the protein to block HIV-1 infection.

## Background

Efficient infection of human primary macrophages, dendritic cells and resting CD4^+^ T-cells by simian immunodeficiency virus (SIV_mac_) requires the accessory protein Vpx [1-6]. Vpx is essential for both SIV infection of primary macrophages and viral pathogenesis *in vivo*[7-10]. Vpx is incorporated into viral particles suggesting that it might be acting immediately after viral fusion [11-14]. Viral reverse transcription is prevented in primary macrophages when cells are infected with either Vpx-deficient SIV_mac_ or HIV-2 [4,15-18]. Interestingly, Vpx also increases the ability of HIV-1 to efficiently infect macrophages, dendritic cells and resting CD4+ T cells when Vpx is incorporated into HIV-1 particles or supplied in trans [1,5,6,19]. Recent work identified SAMHD1 as the protein that blocks infection of SIV∆Vpx, HIV-2∆Vpx and HIV-1 before reverse transcription in macrophages, dendritic cells and resting CD4+ T cells [1,6,20-22]. Mechanistic studies have suggested that Vpx induces the proteasomal degradation of SAMHD1 [20-22]. In agreement, the C-terminal region of SAMHD1 contains a Vpx binding motif, which is important for the ability of Vpx to degrade SAMHD1 [23-26]. SAMHD1 is a dGTP-regulated deoxynucleotide triphosphohydrolase (dNTPase) that decreases the overall cellular levels of dNTPs [27-30].

SAMHD1 is comprised of the sterile alpha motif (SAM) and histidine-aspartic (HD) domains. The HD domain of SAMHD1 is a dGTP-regulated deoxynucleotide triphosphohydrolase that decreases the cellular levels of dNTPs [27-30]. The sole HD domain is sufficient to potently restrict infection by different viruses [31]. The HD domain is also necessary for the ability of SAMHD1 to oligomerize and to bind RNA [31]. The ability of SAMHD1 to block retroviral infection in non-cycling cells, such as macrophages, dendritic cells and resting CD4+ T cells, is controlled by phosphorylation of T592 [32-34]. Phosphorylation of SAMHD1 regulates the capability of SAMHD1 to block HIV-1 infection but not the ability to decrease the cellular levels of dNTPs [33].

In agreement with Goldstone and colleagues, we have established that SAMHD1 is an oligomeric protein in mammalian cells [31,33]; however, the contribution of oligomerization to the ability of SAMHD1 to block HIV-1 infection is not understood. Previous studies have suggested that oligomerization is essential for the enzymatic activity of the HD domain [35]. This work explores the contribution of SAMHD1 oligomerization to HIV-1 restriction, dNTPase activity and nuclease activity. Using the SAMHD1 structure provided by Goldstone and colleagues, we identify key interfacial residues and demonstrate that their mutations disrupt SAMHD1 oligomerization. Recombinant purified oligomerization-deficient SAMHD1 mutants lost their dNTPase but not nuclease activity. In agreement, oligomerization-deficient SAMHD1 mutants immunoprecipitated from mammalian cells lost their dNTPase activity. Remarkably, oligomerization-deficient SAMHD1 variants potently restricted HIV-1 infection. These results suggest that SAMHD1 oligomerization is not required for the ability of the protein to block HIV-1 infection.

## Results

### Mutations of hydrophobic interfacial residues disrupt SAMHD1 oligomerization in mammalian cells

**Figure 1:**
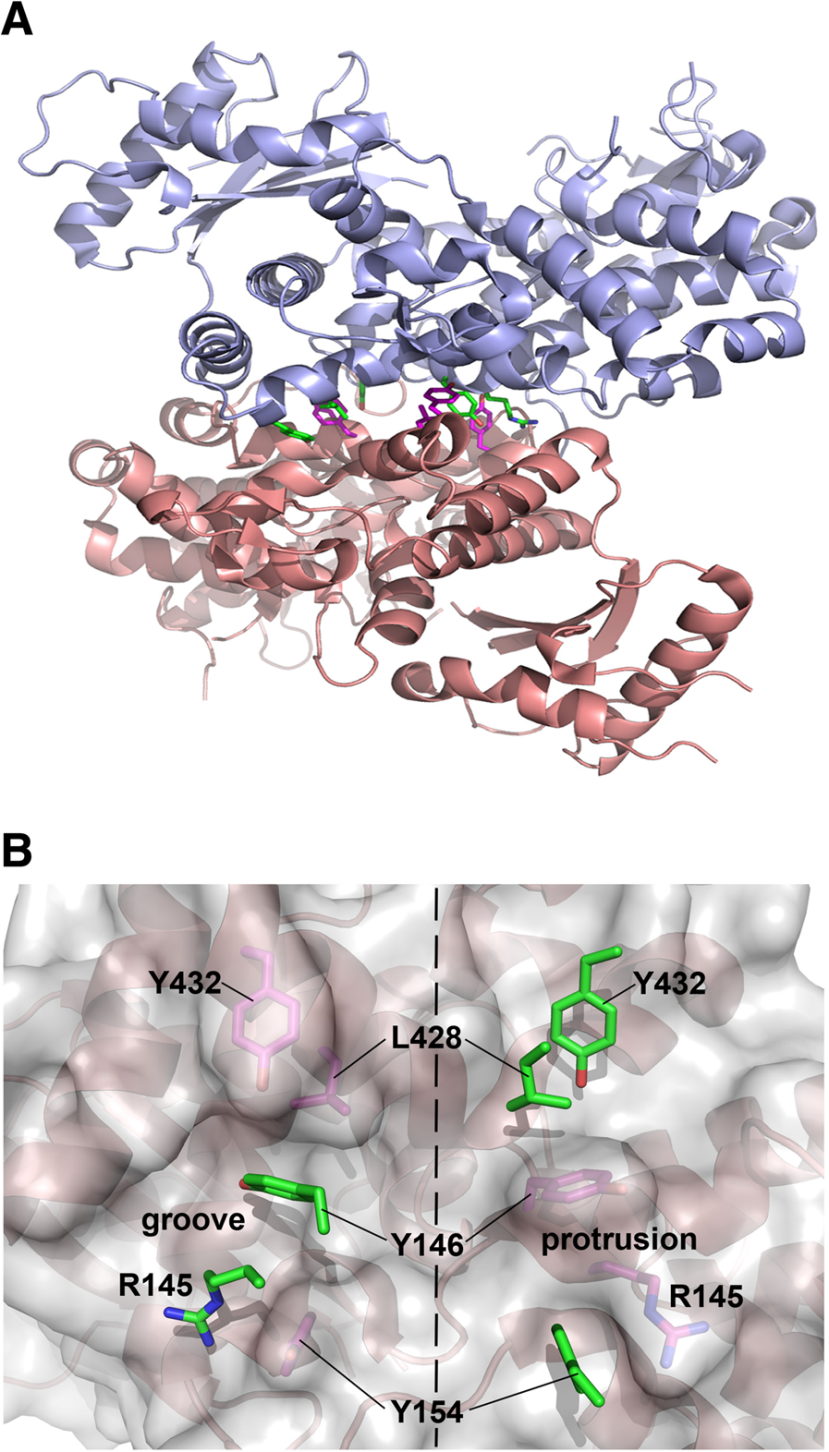
**SAMHD1 dimer interface is stabilized by hydrophobic interactions. (A)** SAMHD1 dimer as observed in the crystal structure (PDB ID: 3U1N) [29]. The extensive dimer interface is stabilized by two hydrophobic patches formed by residues Y146, Y154, L428 and Y432 shown in green and magenta. **(B)** The close-up view showing the packing of the four hydrophobic residues at the interface. The two patches are related by the 2-fold rotational symmetry of the dimer.

The recently discovered restriction factor SAMHD1 blocks infection of HIV-1 and other retroviruses [20,21,27-31,36,37]. In the crystal structure by Goldstone and colleagues the HD domain of the human SAMHD1 appears as a dimer with extensive dimerization interface [29] (Figure 1A). A very similar interface was observed in the structure of EF1143, an HD domain protein from *Enterococcus faecalis*, although the bacterial protein was found to be tetrameric in the crystal [35]. It has been proposed that SAMHD1 also functions as a tetramer [38]. To understand the contribution of oligomerization to the antiviral activity of SAMHD1, we set out to explore the antiviral activity of oligomerization-defective SAMHD1 variants. Inspection of the SAMHD1 crystal structure reveals that the extensive dimer interface is stabilized by two hydrophobic patches formed by residues Y146, Y154, L428 and Y432 (Figure 1B), thus we investigated how mutations of these residues affect SAMHD1 oligomerization and activity.

**Figure 2:**
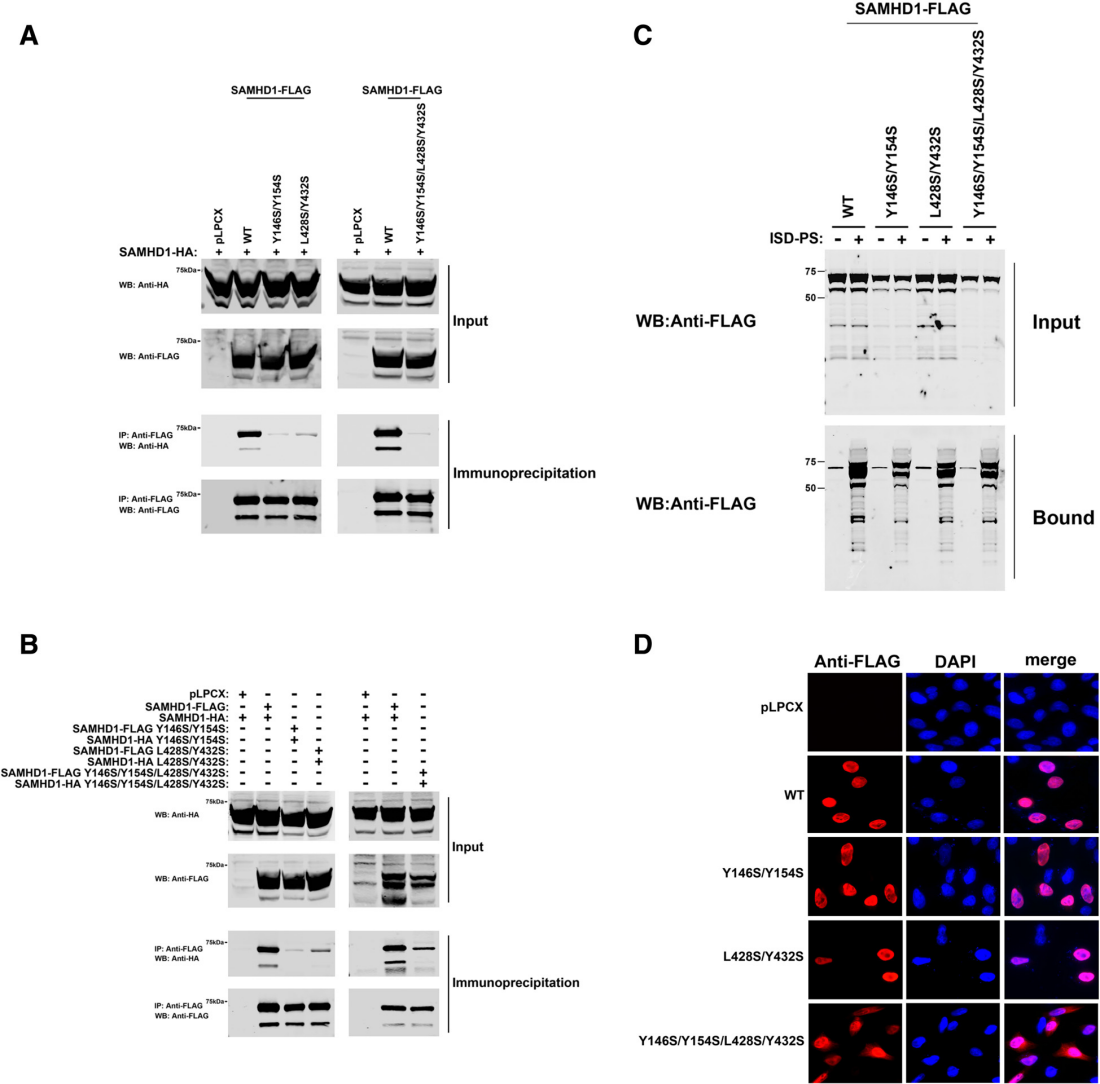
**Oligomerization, RNA binding and intracellular distribution of SAMHD1 variants. (A)** Oligomerization of SAMHD1 variants was tested as previously described [31]. Briefly, human 293 T cells were co-transfected with a plasmid expressing wild type SAMHD1-HA and a plasmid either expressing wild type or mutant SAMHD1-FLAG proteins. Cells were lysed 24 hours after transfection and analyzed by Western blotting using anti-HA and anti-FLAG antibodies (Input). Subsequently, lysates were immunoprecipitated by using anti-FLAG agarose beads. Anti-FLAG agarose beads were eluted using FLAG peptide, and elutions were analyzed by Western blotting using anti-HA and anti-FLAG antibodies (Immunoprecipitation). Similar results were obtained in two independent experiments and representative data is shown. WB, Western blot; IP, Immunoprecipitation; WT, wild type. **(B)** Similar immunoprecipitations were performed by pulling down an HA-tagged variant with its corresponding FLAG-tagged variant. **(C)** The ability of SAMHD1 variants to bind nucleic acids was tested as previously described [31]. Human 293 T cells were transfected with plasmids expressing the SAMHD1 variants were lysed **(Input)** and incubated with the RNA analog ISD-PS immobilized to Strep Tactin Superflow affinity resin. Eluted proteins from the resin were visualized by Western blotting using anti-FLAG antibodies **(Bound)**. Similar results were obtained in three independent experiments and a representative experiment is shown. ISD-PS, interferon-stimulatory DNA sequence containing a phosphorothioate backbone. **(D)** Intracellular distribution of SAMHD1 variants in HeLa cells. HeLa cells expressing the indicated SAMHD1-FLAG variants were fixed and immunostained using antibodies against FLAG (red) as previously described [31,44]. Cellular nuclei were stained by using DAPI (blue). Image quantification for three independent experiments is shown in Additional file 1.

To test the hypothesis that residues in the hydrophobic patches stabilized the dimer interface, we tested the ability of these mutants to oligomerize by using our previously described oligomerization assay [31]. As shown in Figure 2A and Table 1, FLAG-tagged SAMHD1 variants Y146S/Y154S, L428S/Y432S and Y146S/Y154S/L428S/Y432S lost the ability to oligomerize with the HA-tagged wild-type SAMHD1 (mutant association to wild type), suggesting that these variants are no longer able to form oligomers. We also tested the ability of each FLAG-tagged variant to interact with its corresponding HA-tagged mutant (Figure 2B and Table 1) (mutant self-association). These results showed that the SAMHD1 oligomerization-defective variants were not able to interact with themselves.

**Table 1:** SAMHD1 oligomerization variants

**SAMHD1 variant**	**HIV-1 Restriction**^**a**^	**Oligomerization**^**b**^	**RNA Binding**^**c**^	**Localization**^**d**^	**Cellular dNTP Level**^**e**^	**Association with WT ± SD**^**f**^	**Self-association ± SD**^**g**^
WT	+	+	+	N	Low	100	100
Y146S/Y154S	+	-	+	N	Low	10.15 ± 1.48	2.46 ± 0.69
L428S/Y432S	+	-	+	N	Low	25.9 ± 9.19	16.04 ± 1.50
Y146S/Y154S/L428S/Y432S	ND	-	+	N/C	Low	1.4 ± 0.25	22.04 ± 7.70

^**a**^HIV-1 restriction was measured by infecting U937 cells stably expressing the indicated SAMHD1 variants with HIV-1-GFP. After 48 hours, the percentage of GFP-positive cells (infected cells) was determined by flow cytometry.

^**b**^Oligomerization of the different SAMHD1 variants was determined by measuring the ability of the SAMHD1-FLAG variant to interact with wild type SAMHD1-HA variant, as described [31]. “ + ” indicates 100% oligomerization, which corresponds to the amount of wild type SAMHD1-HA that interacts with wild type SAMHD1-FLAG. “ - ” indicates the absence of oligomerization.

^**c**^SAMHD1-FLAG variants were assayed for their ability to bind the double-stranded RNA analog ISD-PS, as described [31]. “+” indicates the RNA binding achieved by wild type SAMHD1.

^**d**^Subcellular localization of the different SAMHD1 variants in HeLa cells was performed as described [31]. “N” indicates nuclear localization; “N/C” indicates nuclear and cytoplasmic localization.

^**e**^The cellular dATP levels of PMA-treated U937 cells stably expressing the different SAMHD1 variants were determined by primer extension as described [31]. “Low” indicates similar to the dATP levels observed in PMA-treated U937 cells stably expressing wild type SAMHD1.

^**f**^WT and SAMHD1-FLAG variants were assayed for association with wild-type SAMHD1-HA as described [31]. Percentages are an average of two independent experiments. The percentage represents the fraction of the SAMHD1 variant coprecipitated with wild-type SAMHD1 relative to the amount of wild-type SAMHD1 coprecipitated with itself.

^**g**^WT and SAMHD1-FLAG variants were assayed for association with wild-type and variant SAMHD1-HA as described [33]. Percentages are an average of two independent experiments. The percentage represents the fraction of the SAMHD1 variant coprecipitated with itself relative to the coprecipitation of wild-type SAMHD1 with itself.

To indirectly rule out the possibility that SAMHD1 oligomerization-defective variants are not misfolded proteins, we tested for the ability of these variants to bind RNA (Figure 2C and Table 1), as described [31]. For this purpose we tested the ability of SAMHD1 to interact with the interferon-stimulatory DNA sequence containing a phosphorothioate backbone (ISD-PS), which is an RNA analog [31,39]. As shown in Figure 2C, all tested SAMHD1 variants were able to interact with the RNA analog ISD-PS. These results indicated that oligomerizaton is not required for the ability of SAMHD1 to bind RNA (Table 1). Next we tested the ability of the SAMHD1 variants to localize to the nuclear compartment (Figure 2D). SAMHD1 variants Y146S/Y154S and L428S/Y432S exclusively localized to the nuclear compartment (Figure 2D and Table 1). By contrast, image quantification of the SAMHD1 variant Y146S/Y154S/L428S/Y432S showed that this variant does not exhibit complete nuclear localization suggesting that this particular variant has lost a function or its partially misfolded (Figure 2D and Additional file 1). Because the SAMHD1 variant Y146S/Y154S/L428S/Y432S has lost nuclear localization, we will no longer pursue its analysis.

### The SAMHD1 variant Y146S/Y154S loses dGTP-dependent tetramerization *in vitro*

To get a more refined mechanistic understanding of the effect of the interfacial SAMHD1 mutations we performed in vitro comparative studies of SAMHD1 oligomerization. The HD domain construct of human SAMHD1 used by Goldstone and colleagues in the crystallographic studies (residues 120–626) [29] lacks several N-terminal residues that are important for the binding of dGTP at the allosteric site, as observed in the bacterial HD domain homologue to SAMHD1 [35]. Therefore, we used an extended construct that comprises SAMHD1 residues 109–626 for our *in vitro* studies.

**Figure 3:**
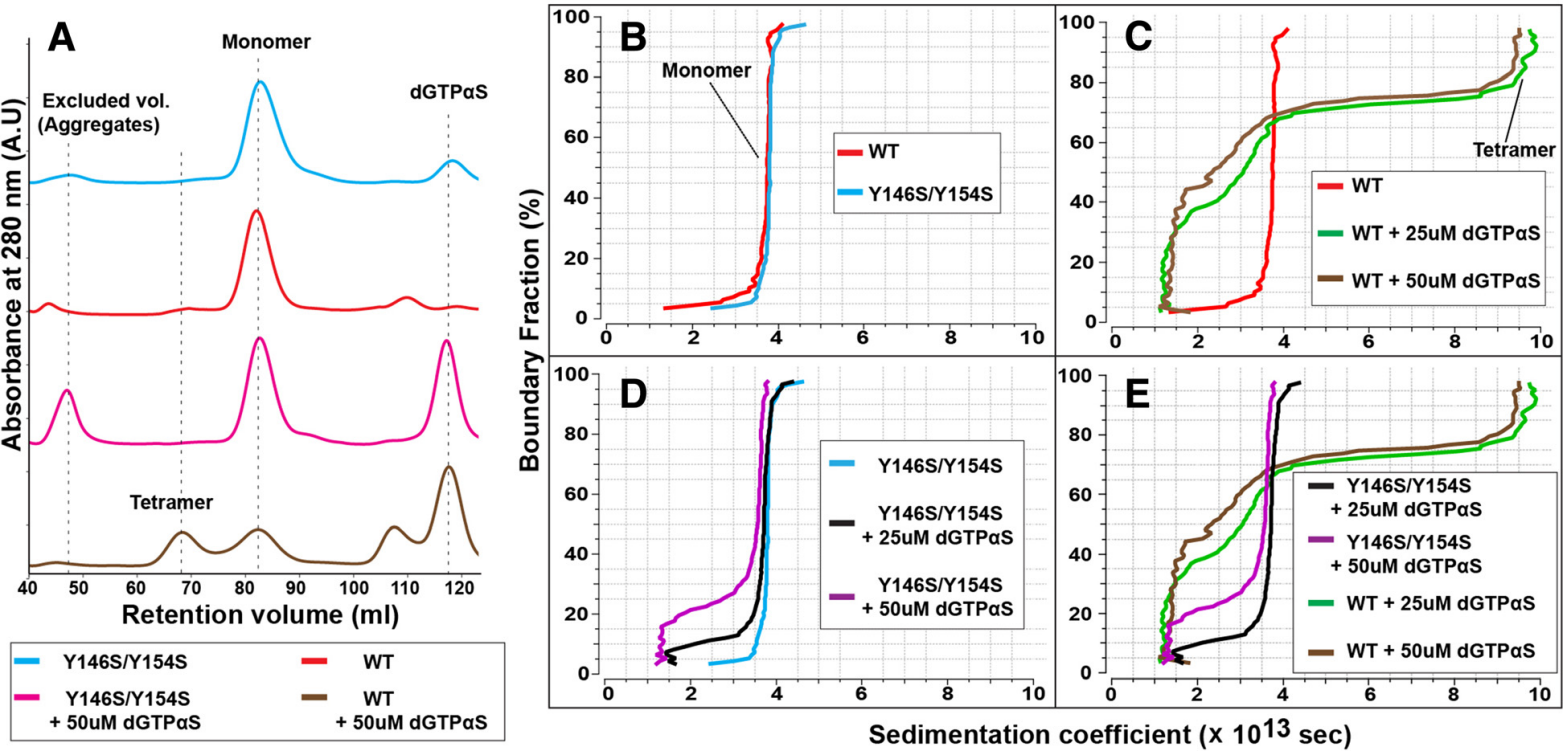
**Analysis of SAMHD1 oligomerization by size-exclusion chromatography and analytical ultracentrifugation. (A)** Size exclusion chromatograms of the wild type (WT) and Y146S/Y154S 109–626 SAMHD1 constructs before and after incubation with dGTPαS. **(B-E)** Comparison plots of the diffusion-corrected integral sedimentation coefficient distributions obtained from a van Holde – Weischet analysis. WT and Y146S/Y154S without dGTPαS **(B)**; WT incubated with 0, 25 and 50 μM dGTPαS **(C)**; Y146S/Y154S incubated with 0, 25 and 50 μM dGTPαS **(D)**; comparison of WT and Y146S/Y154S distributions following incubation with dGTPαS **(E)**. Similar results were obtained in three independent experiments and a representative experiment is shown.

Size-exclusion chromatography of the purified wild type and Y146S/Y154S variant of the SAMHD1 construct 109–626 were performed on the HiLoad 16/60 Superdex 200 media (GE Life Sciences), and showed that both proteins elute as single peaks at the retention volume of approximately 82 mL indicating that both recombinant proteins are predominantly monomeric in solution (Figure 3A). Following incubation of the proteins with dGTPαS, a dGTP analog that is hydrolyzed by SAMHD1 at a slower rate, size exclusion chromatography revealed an additional peak at ~69 mL in the chromatogram of the wild type protein, which is absent in the Y146S/Y154S sample. This peak is distinct from the high molecular weight aggregates, which elute in the excluded volume (42–45 mL) of the HiLoad 16/60 Superdex 200 column. Most likely the 69 mL peak corresponds to the previously reported tetrameric form of the HD domain [38].

The effect of dGTPαS incubation on the oligomeric state of the protein was investigated using sedimentation velocity as described in [40]. Diffusion-corrected van Holde – Weischet sedimentation coefficient distributions [41] of the purified proteins (Figure 3B) revealed mono-disperse species with sedimentation coefficient close to 4. Additional 2DSA-Monte Carlo analysis [42,43] reports a frictional ratio of ~1.5, which corresponds to a molecular weight of ~60 kDa, in agreement with a monomeric state. Incubation of wild type monomeric SAMHD1 with dGTPαS induced the formation of high molecular weight species; this oligomer sediments at approximately 9.7 s consistent with a 240 kDa tetramer with a frictional ratio of 1.5 (Figure 3C and E). By contrast, dGTPαS had no effect on the oligomerization state of the Y146S/Y154S variant (Figure 3D-E), which is in agreement with the results obtained by size-exclusion chromatography. In all samples, we observed the appearance of a low sedimentation component (< 2) most likely the result of dGTPαS absorption at 280 nm. Collectively, this data demonstrates that the recombinant wild type HD domain of SAMHD1 can form a tetramer in a dGTP-dependent manner, and that tetramerization is disrupted by the Y146S/Y154S mutation.

### Y146S/Y154S mutation disrupts the deoxynucleotide triphosphohydrolase (dNTPase) but not the nuclease activity of SAMHD1

To understand the contribution of dGTP-mediated tetramerization to SAMHD1 enzymatic activity, we investigated the dNTPase and nuclease activity of Y146S/Y154S and wild type SAMHD1 proteins.

**Figure 4:**
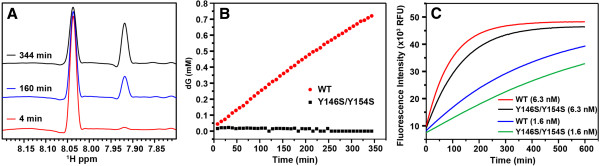
**Effect of Y146S/Y154S mutation on the dNTPase and nuclease activity of SAMHD1 in vitro. (A)** The region of the ^1^H NMR spectrum with the signal of the H8 proton of the guanine base. The peak is shifted from 8.04 ppm to 7.92 ppm upon hydrolysis of dGTP to deoxyguanosine. The three spectra were collected 4, 160 and 344 min after addition of 2 μM wild type (WT) 109–626 SAMHD1 to a sample containing 2 mM dGTP. **(B)** Concentration of deoxyguanosine released in SAMHD1-catalyzed dGTP hydrolysis as a function of time. **(C)** The nuclease activity of wild type and Y146S/Y154S SAMHD1 proteins was measure using a nuclease activity assay. Briefly the different proteins were incubated with a single stranded DNA (ssDNA) containing a 5′ FAM label and a 3′ BHQ1 black hole quencher. The fluorescence of the ssDNA substrate containing a 5′ FAM label and a 3′ BHQ1 black hole quencher is increased more than 6 fold after the ssDNA is cleaved. Plots of total FAM fluorescence measured as a function of time reveal that Y146S/Y154S mutation has only a modest effect on SAMHD1 nuclease activity.

To study the dNTPase activity, we used an NMR-based dGTP hydrolysis assay to monitor the dNTPase activity of SAMHD1 (Figure 4A). The H8 proton of the guanine base appears as a narrow singlet peak at 8.04 ppm in the ^1^H NMR spectrum of dGTP. This signal is shifted to 7.92 ppm upon hydrolysis of dGTP to deoxyguanosine, and can thus be used to monitor SAMHD1-catalyzed dGTP hydrolysis reaction in real time (Figure 4A). The assay revealed that the wild type construct hydrolyzed dGTP whereas the activity of the Y146S/Y154S mutant was virtually undetectable (Figure 4B). 

Subsequently, we tested the nuclease activity of the two SAMHD1 constructs using a quenched fluorescent single-stranded DNA substrate as described in Methods*.* The measured activity of the Y146S/Y154S variant is slightly lower when compared to the nuclease activity of the wild type protein (Figure 4C). These results indicated that in contrast to the dNTPase activity, the nuclease activity of SAMHD1 is not subject to allosteric regulation via dGTP-dependent tetramerization.

### Y146S/Y154S and L428S/Y432S SAMHD1 variants disrupt the dNTPase activity of full-length SAMHD1 immunoprecipitated from mammalian cells

**Figure 5:**
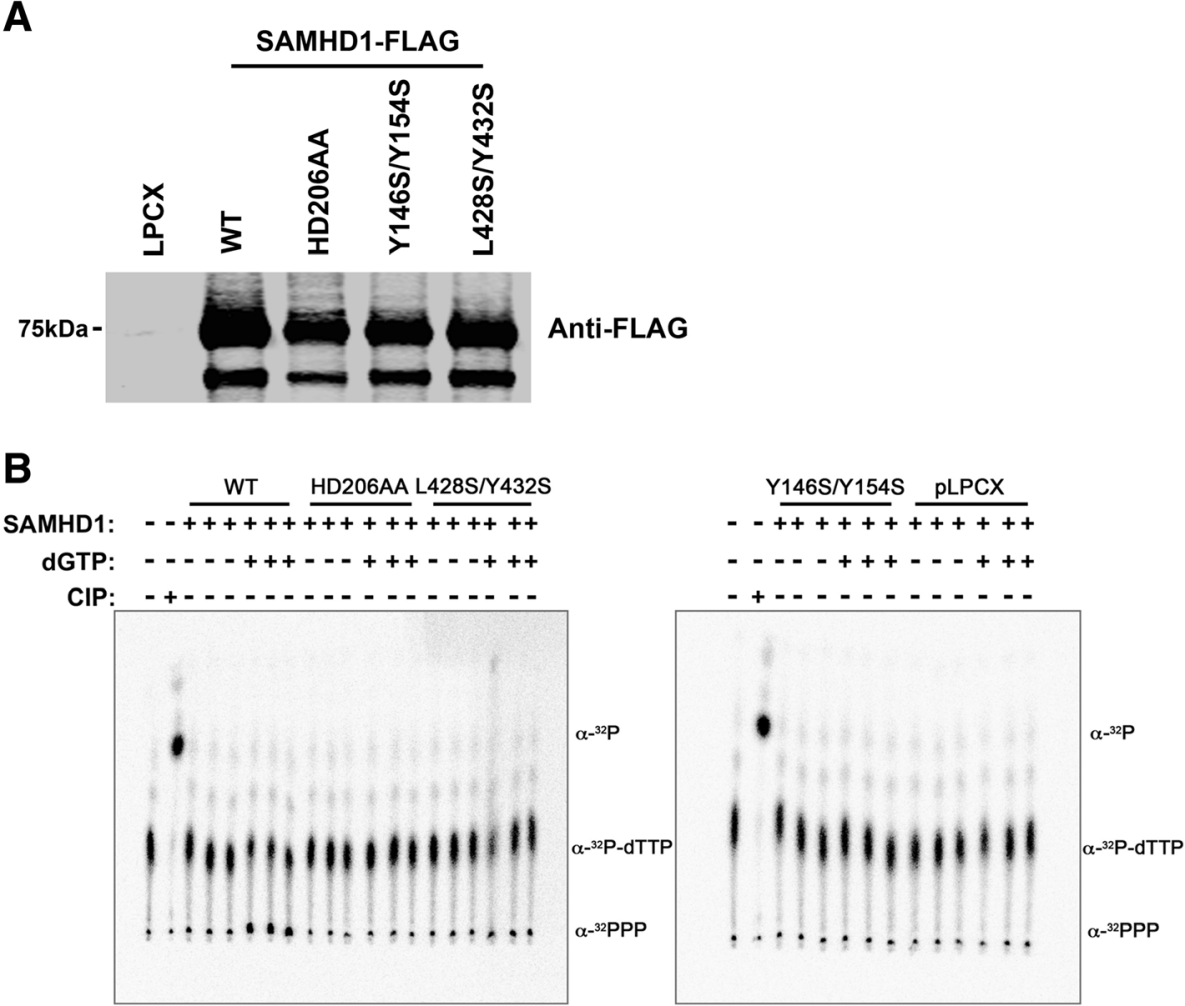
**dNTPase activity of SAMHD1 oligomerization variants immunoprecipitated from mammalian cells.** The indicated FLAG-tagged SAMHD1 variants were immunoprecipitated **(A)**, and tested for their ability to hydrolyze α - ^32^P-TTP to dT and α - ^32^PPP, in the presence of the allosteric activator dGTP. Reactions products were separated using thin-layer chromatography using polyethyleneimine cellulose in order to determine the amount of hydrolyzed α - ^32^PPP **(B)**. As a control, we have included the mutant HD206AA, which is a SAMHD1 protein defective in the active site of the HD domain. The results of three independent enzymatic reactions per treatment are shown. WT, wild type; CIP, calf intestine phosphatase.

To directly analyze the dNTPase activity of SAMHD1 full-length variants, we tested the ability of immunoprecipitated SAMHD1 variants (Figure 5A) to hydrolyze α-^32^P-TTP to dT and α-^32^PPP, in the presence of the allosteric activator dGTP. For this purpose we incubated the indicated SAMHD1 variant in the presence of radio-labeled α-^32^P-TTP. Reaction products were separated using thin-layer chromatography in order to determine the amount of hydrolyzed α-^32^PPP (Figure 5B), as previously shown [31,33]. In agreement with our results using bacterially purified protein, immunoprecipitated Y146S/Y154S and L428S/Y432S SAMHD1 variants lost dNTPase activity when compared to wild type SAMHD1 (Figure 5B). As expected, the SAMHD1 variant HD206AA completely lost dNTPase activity [31,33]. These results suggested that mutants that lost the ability to form tetramers in a dGTP-dependent manner were also defective in their dNTPase activity.

### Ability of SAMHD1 variants to restrict HIV-1 infection

**Figure 6:**
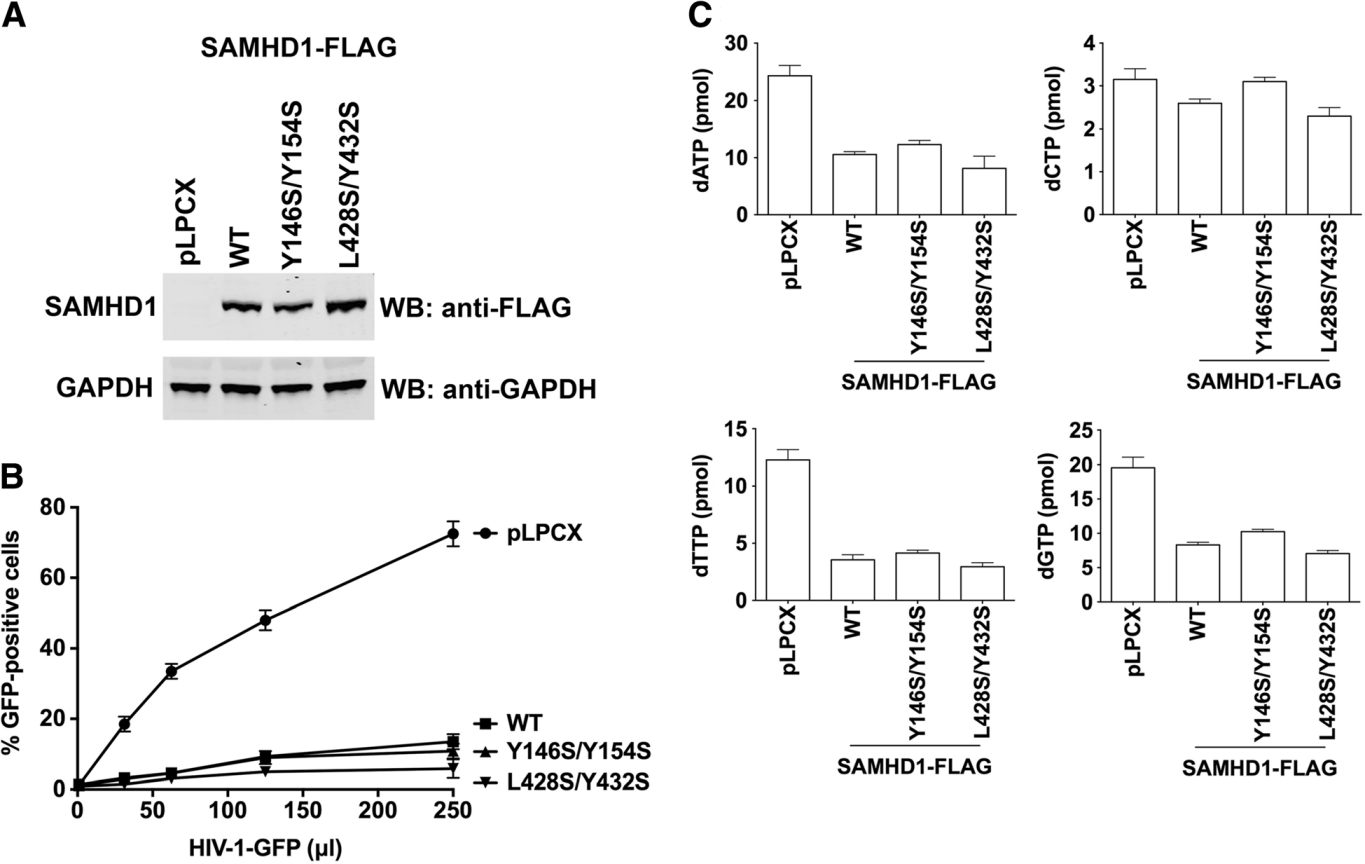
**Ability of SAMHD1 oligomerization variants to restrict HIV-1.** PMA-treated human monocytic U937 cells stably expressing the indicated SAMHD1 variant **(A)** were challenged with increasing amounts of HIV-1-GFP **(B)**. As control, U937 cells stably transduced with the empty vector LPCX were challenged with HIV-1-GFP. Similar results were obtained in three independent experiments and a representative experiments is shown. **(C)** Quantification of dNTP levels on PMA-treated U937 cells expressing the indicated SAMHD1 variants was performed by a primer extension methodology, as previously described [31].

To understand whether dGTP-dependent tetramerization contributes to the antiretroviral properties of SAMHD1, we tested the ability of dGTP-dependent tetramerization-defective SAMHD1 variants to restrict HIV-1 infection. For this purpose, we stably expressed the indicated SAMHD1 variants in human monocytic U937 cells (Figure 6A), and tested them for the ability to block HIV-1 infection. PMA-treated U937 cells stably expressing SAMHD1 variants were challenged with increasing amounts of HIV-1 virus expressing GFP as a reporter of infection (Figure 6B and Table 1). Remarkably, SAMHD1 variants that lost dGTP-dependent tetramerization potently restricted HIV-1 infection. These results suggested that SAMHD1 dGTP-dependent tetramerization is not required for the ability of SAMHD1 to block infection.

Because expression of SAMHD1 in U937 cell decreases the cellular levels of deoxynucleotides (dNTPs), we measured the cellular levels of dNTPs in U937 cells expressing the different SAMHD1 variants, as previously described (Figure 6C and Table 1) [31]. Interestingly, SAMHD1 oligomerization variants decreased the cellular levels of dNTPs (Figure 6C and Table 1) indicating that the dNTPase activity of SAMHD1 in mammalian cells may be upregulated by a mechanism that does not depend on tetramerization and dGTP binding.

### Vpx-mediated degradation of SAMHD1 variants

**Figure 7:**
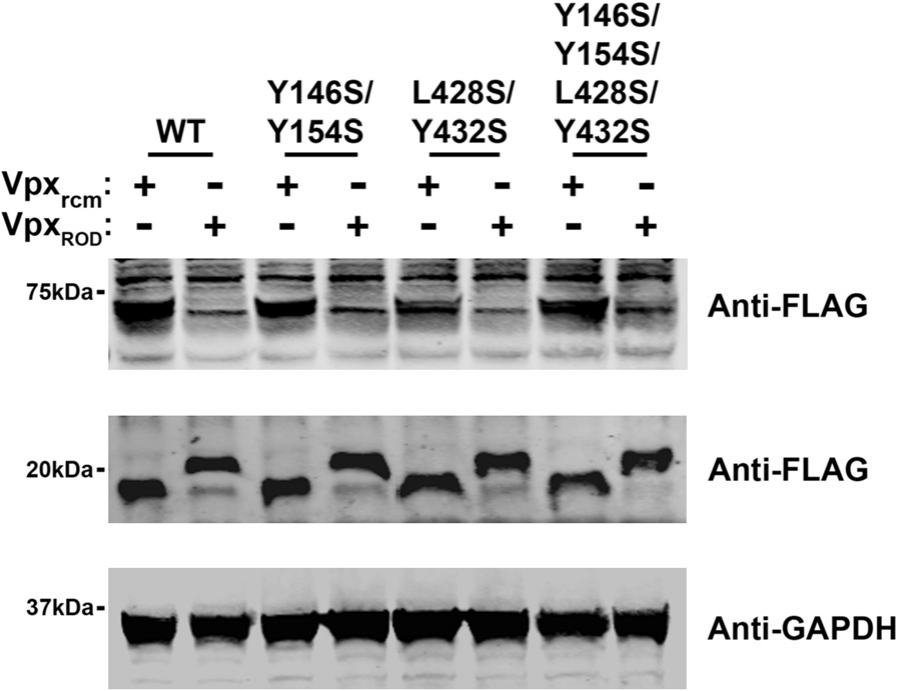
**Vpx-induced degradation of SAMHD1 variants.** HeLa cells were cotransfected with plasmids allowing expression of SAMHD1-FLAG variants and the Vpx protein of HIV-2_ROD_ (Vpx_ROD_) or the Vpx protein of SIVrcm (Vpx_rcm_), as described [44]. Thirty-six hours post-transfection the cells were harvested, and the expression levels of SAMHD1 and Vpx were analyzed by Western blot using anti-FLAG antibodies. As a loading control, cell extracts were Western blotted using antibodies against GAPDH. Similar results were obtained in three independent experiments and a representative experiment is shown.

Finally, we explored the ability of Vpx from HIV-1-ROD (Vpx_rod_) to degrade SAMHD1 oligomerization-defective variants, as previously described [44]. As shown in Figure 7, tetramerization-defective SAMHD1 variants were degraded by Vpx_rod_. As a control, we used the Vpx protein from red-capped mangabeys (Vpx_RCM_), which does not induce the degradation of SAMHD1. These results indicated that dGTP-induced tetramerization is not required for the ability of Vpx to degrade SAMHD1.

## Discussion

Overall, the work presented here analyzes the contribution of oligomerization to the different functions of SAMHD1. Close analysis of the interfacial residues in the structure presented by Goldstone and colleagues revealed four residues (Y146, Y154, L428 and Y432) that might be stabilizing the hydrophobic interactions between the monomers in the dimer structure [29]. To test this hypothesis we tested the ability of the double mutants Y146S/Y154S and L428S/Y432S to form oligomers. Using our oligomerization assay that utilizes proteins extracted from mammalian cells [31], we found that SAMHD1 variants Y146S/Y154S and L428S/Y432S completely lost their ability to form oligomers. In agreement, the recombinant Y146S/Y154S variant of the HD domain construct (SAMHD1 residues 109–626) lost its dGTP-dependent tetramerization ability when compared to wild type protein, as measured by gel filtration and analytical ultracentrifugation. These results show that hydrophobic interfacial residues Y146, Y154, L428 and Y432 are critical for the dGTP-dependent tetramerization ability of SAMHD1.

Next we explored the contribution of oligomerization to the described enzymatic activities of SAMHD1. The HD domain of SAMHD1 exhibits dNTPase and nuclease activity [28-31,45]. Interestingly, SAMHD1 oligomerization-defective variants lost their dNTPase activity when SAMHD1 proteins were prepared in bacteria or in mammalian cells. These results suggested that tetramerization is important for dNTPase activity, as previously suggested [29,35,38]. In contrast, the nuclease activity of the Y146S/Y154S oligomerization-defective SAMHD1 variant was not significantly perturbed. Overall, these findings suggested that dGTP-dependent SAMHD1 tetramerization is important for dNTPase but not nuclease activity. These results are interesting in the light of the new discovery that SAMHD1 exhibit nuclease activity [45], suggesting that RNAase might be part of the mechanism by which SAMHD1 blocks HIV-1 infection.

We found that SAMHD1 variants that are defective for dGTP-dependent tetramerization potently blocked HIV-1 infection when compared to wild type SAMHD1, which suggested that oligomerization is not required for the antiretroviral properties of SAMHD1. Surprisingly, SAMHD1 oligomerization-deficient mutants were able to decrease the dNTP cellular levels when compared to wild type SAMHD1. These results suggest that the dNTPase activity of SAMHD1 might be regulated in cells by a yet unknown mechanism that does not require tetramerization. Another possibility is that SAMHD1 mutants that are strongly oligomerization-deficient in our in-vitro and immunoprecipitation assays described here, are still capable of forming tetramers when inside mammalian cells through interaction with other factors or some other compensatory mechanism. Future experiments will determine whether dNTPase and/or nuclease activities are required to block HIV-1 infection.

## Conclusions

These results suggested that SAMHD1 oligomerization is not required for the ability of the protein to block HIV-1 infection.

## Methods

### Generation of U937 cells stably expressing SAMHD1 variants

Retroviral vectors encoding wild type or mutant SAMHD1 proteins fused to FLAG were created using the LPCX vector (Clontech). Recombinant viruses were produced in 293 T cells by co-transfecting the LPCX plasmids with the pVPack-GP and pVPack-VSV-G packaging plasmids (Stratagene). The pVPack- VSV-G plasmid encodes the vesicular stomatitis virus G envelope glycoprotein, which allows efficient entry into a wide range of vertebrate cells [46]. Transduced human monocytic U937 cells were selected in 0.4 mg/ml puromycin (Sigma).

### Infection with HIV-1 expressing the green fluorescent protein (GFP)

HIV-1 expressing GFP, pseudotyped with the VSV-G glycoprotein, were prepared as described [47]. For infections, 6 × 10^4^ cells seeded in 24-well plates were either treated with 10 ng/ml phorbol-12-myristate-3-acetate (PMA) or DMSO for 16 h. PMA stock solution was prepared in DMSO at 250 mg/ml. Subsequently, cells were incubated with HIV-1-GFP for 48 h at 37°C. The percentage of GFP-positive cells was determined by flow cytometry (Becton Dickinson). Viral stocks were titrated by serial dilution on dog Cf2Th cells.

### SAMHD1 oligomerization assay

Approximately 1.0 × 10^7^ human 293 T cells were cotransfected with plasmids encoding SAMHD1 variants tagged with FLAG and HA. After 24 h, cells were lysed in 0.5 ml of whole-cell extract (WCE) buffer [50 mM Tris (pH 8.0), 280 mM NaCl, 0.5% IGEPAL, 10% glycerol, 5 mM MgCl2, 50 μg/ml ethidium bromide, 50 U/ml benzonase tail (Roche)]. Lysates were centrifuged at 14,000 rpm for 1 h at 4°C. Post-spin lysates were then pre-cleared using protein A-agarose (Sigma) for 1 h at 4°C; a small aliquot of each of these lysates was stored as input. Pre- cleared lysates containing the tagged proteins were incubated with anti-FLAG-agarose beads (Sigma) for 2 h at 4°C. Anti-FLAG- agarose beads were washed three times in WCE buffer, and immune complexes were eluted using 200 mg of FLAG tripeptide/ml in WCE buffer. The eluted samples were separated by SDS-PAGE and analyzed by Western blotting using either anti-HA or anti-FLAG antibodies (Sigma).

### Nucleic-acid binding assay

Nucleic-acid binding assay was performed as previously described [31,39]. In brief, the synthetic DNA phosphorothioate-containing interferon-stimulatory DNA (ISD-PS), which is an RNA analog, was synthesized with a 50-biotin tag using the following primers:

ISD sense 5′-tacagatctactagtgatctatgactgatctgtacatgatctaca-3′,

ISD antisense 5′-tgtagatcatgtacagatcagtcatagatcactagtagatctgta-3′.

Sense and antisense primers were incubated at 65°C for 20 min, and primers were allowed to anneal by cooling down to room temperature. Annealed primers were immobilized on an Ultralink Immobilized Streptavidin Plus Gel (Pierce). Cells were lysed using TAP lysis buffer (50 mM Tris pH 7.5, 100 mM NaCl, 5% glycerol, 0.2% NP-40, 1.5 mM MgCl2, 25 mM NaF, 1 mM Na3VO4, protease inhibitors) and lysates were cleared by centrifugation. Cleared lysates (Input) were incubated with immobilized nucleic acids at 4°C on a rotary wheel for 2 h in the presence of 10 mg/ml of Calf-thymus DNA (Sigma) as a competitor. Unbound proteins were removed by three consecutive washes in TAP lysis buffer. Bound proteins to nucleic acids (Bound) were eluted by boiling samples in SDS sample buffer (63 mM Tris–HCl, 10% Glycerol 2% SDS, 0.0025% Bromophenol Blue) and analyzed by Western blot- ting using anti-FLAG antibodies (Sigma).

### In vitro oligomerization assays

WT and Y146S/Y154S variant of the strep-tagged HD domain construct of human SAMHD1 (residue 109–626) were expressed in BL21(DE3) E.coli using a pET expression vector. Protein was purified by affinity chromatography [29].

SAMHD1 constructs at 8 μM concentration were incubated with or without 50 μM dGTPαS for 4 days at 4C. After the incubation the samples were analyzed by size-exclusion chromatography using a HiLoad 16/60 Superdex 200 column. (GE Life Sciences).

Sedimentation velocity analytical ultracentrifugation was performed on a Beckman XLA analytical ultracentrifuge using an AN50 Ti rotor with standard Epon 2-channel centerpieces. The samples were spun at 40000 rpm for ~ 12 hrs at 20 C. 25 scans measuring absorbance at 280 nm were collected. The van Holde – Weischet and 2DSA-Monte Carlo analysis was performed using Ultrascan 3 as described elsewhere [40-43].

### In vitro dNTPase and nuclease assays

dNTPase. SAMHD1 was buffer-exchanged into NMR buffer (50 mM d11-Tris, pH 7.4, 50 mM NaCl, 5 mM MgCl2, 50uM Zn2+). NMR samples were prepared as follows: 2 mM dGTP, 2uM SAMHD1, 10% D2O in NMR buffer. ^1^H 1D NMR spectra were recorded every 4 min using SampleJet on a 500 MHz Bruker spectrometer equipped with TCI cryo-probe. NMR 1D spectra were processed using NMRPipe.

Nuclease. A quenched fluorescent single-stranded DNA substrate was used to measure the nuclease activity of SAMHD1 HD domain constructs. The single-stranded 45-base DNA oligo 5′-tacagatctactagtgatctatgactgatctgtacatgatctaca-3′ was ordered from MWG operon with 5′-FAM and 3′-BHQ1 modifications. The substrate (100 μM) and the enzyme (12.5 μM and 3.25 μM) stocks were prepared in the assay buffer (50 mM tris, pH 7.4, 5 mM MgCl2, 50 uM Zn2+ and 50 mM NaCl). 20 μL of the substrate stock was mixed with 20 μL of the enzyme stock in a 384-well microplate and the fluorescence signal measured on a Biotek Synergy 2 microplate reader using 485/20 excitation and 528/20 emission filters. The fluorescence intensities were plotted as a function of the reaction time.

### Determination of dNTPs cellular levels

2 × 10^6^ to 3 × 10^6^ cells werecollected for each cell type. Cells were washed twice with 1x PBS, pelleted and resuspended in ice cold 65% methanol. Cells were vortexed for 2 min and incubated at 95°C for 3 min. Cells were centrifuged at 14000 rpm for 3 min and the supernatant was transferred to a new tube for the complete drying of the methanol in a speed vac. The dried samples were resuspended in molecular grade dH2O. An 18-nucleotide primer labeled at the 5 end with 32 P (5-′GTCCCTGTTCGGGCGCCA-3) was annealed at a 1:2 ratio to four different 19-nucleotide templates (5′-NTGGCGCCCGAACAGGGAC-3′), where'N’ represents the nucleotide variation at the 5′ end. Reaction condition contains 200 fmoles of template primer, 2 ml of 0.5 mM dNTP mix for positive control or dNTP cell extract, 4 ml of excess HIV-1 RT, 25 mM Tris–HCl, pH 8.0, 2 mM dithiothreitol, 100 mM KCl, 5 mM MgCl_2_, and 10 μM oligo(dT) to a final volume of 20 mL. The reaction was incubated at 37°C for 5 min before being quenched with 10 mL of 40 mM EDTA and 99% (vol/vol) formamideat 95°C for 5 min.The extended primer products were resolved on a 14% urea–PAGE gel and analyzed using a phosphoimager. The extended products were quantified using QuantityOne software to quantify percent volume of saturation. The quantified dNTP content of each sample was accounted for based on its dilution factor, so that each sample volume was adjusted to obtain a signal within the linear range of the assay.

### Immunofluorescence microscopy

Transfections of cell monolayers were performed using Lipofectamine Plus reagent (Invitrogen), according to the manufacturer’s instructions. Transfections were incubated at 37°C for 24 h. Indirect immunofluorescence microscopy was perfomed as previously described [44]. Transfected monolayers grown on coverslips were washed twice with PBS1X (137 mM NaCl, KCl 2.7 mM, Na_2_HPO_4_^.^2H_2_O 10 mM, KH_2_PO_4_ mM) and fixed for 15 min in 3.9% paraformaldehyde in PBS1X. Fixed cells were washed twice in PBS1X, permeabilized for 4 min in permeabilizing buffer (0.5% Triton X-100 in PBS), and then blocked in PBS1X containing 2% bovine serum albumin (blocking buffer) for 1 h at room temperature. Cells were then incubated for 1 h at room temperature with primary antibodies diluted in blocking buffer. After three washes with PBS, cells were incubated for 30 min in secondary antibodies and 1 mg of DAPI (49, 69-diamidino-2-phenylindole)/ml. Samples were mounted for fluorescence microscopy by using the ProLong Antifade Kit (Molecular Probes, Eugene, OR). Images were obtained with a ZeissObserver Z1 microscope using a 63x objective, and deconvolution was performed using the software AxioVision V4.8.1.0 (Carl Zeiss Imaging Solutions).

### Assay to determine dNTPase activity of SAMHD1 by thin-liquid chromatography

Wild type and mutant SAMHD1 proteins immunoprecipiated from mammalian cells were incubated with or without 100 μM dGTP, 500 μM dTTP and 0.25 μl α32P-dTTP (PerkinElmer) in SAMHD1 reaction buffer (50 mM Tris–HCl pH 8, 50 mM KCl, 5 mM MgCl_2_, 0.1% Triton-X 100) in a 17.5 μl final volume. Reactions were initiated by addition of SAMHD1, incubated for 1 h at 37°C, and terminated by incubation for 10 min at 70°C. The no enzyme control reaction and the antarctic phosphatase reaction contained dGTP. The antarctic phosphatase reaction (2 ul, New England BioLabs) was used to show the mobility of monophosphates on the plate as a comparison to triphosphate mobility. Reactions were spotted (0.5 μl) on a TLC PEI Cellulose F plate (EMD Chemicals) and separated in a 0.8 M LiCl solvent. Product formation was analyzed on a Bio-Rad Personal Molecular Imager.

## Competing interests

The authors declare no competing interests.

## Authors’ contributions

ABN, JVC, TEW, AB, ZW, BD, LN, BK, SA, CK performed experiments. DI modeled the dimer interface. FDG design experiments and wrote the manuscript. All authors read and approved the final manuscript.

## Supplementary Material

Additional file 1 **Image quantification.** Cells were scored visually for nuclear and cytoplasmic distribution. In every experiment two hundred cells were counted. The analysis of the distribution was performed in human HeLa cells. Click here for file
