# Supplementary figures and images for: Contribution of oligomerization to the anti-HIV-1 properties of SAMHD1
Source: Retrovirology. 2013 Nov 12;10:131. doi: 10.1186/1742-4690-10-131 (PMC3882887; doi:10.1186/1742-4690-10-131)

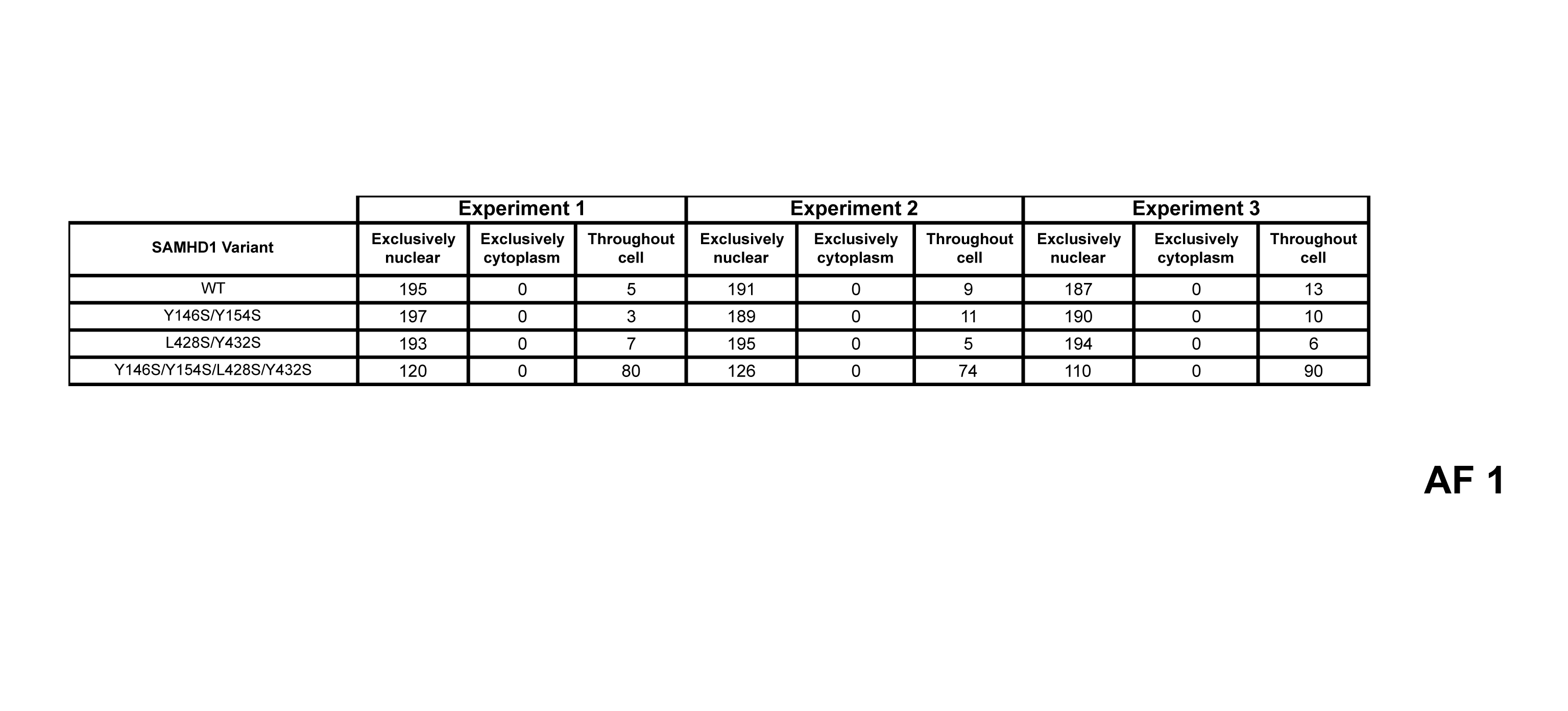

Supplement: Additional file 1 — Image quantification. Cells were scored visually for nuclear and cytoplasmic distribution. In every experiment two hundred cells were counted. The analysis of the distribution was performed in human HeLa cells. [file 1742-4690-10-131-S1.jpeg]
